# Immune-Related Adverse Events of Cemiplimab Therapy in Advanced Cervical Cancer—Data from the Polish–Czech Cervical Cancer Immunotherapy Group (PCCIG-01) with a Review of the Literature

**DOI:** 10.3390/antib15030042

**Published:** 2026-05-18

**Authors:** Radosław Łupkowski, Karolina Górniak, Maja Lisik-Habib, Ewa Burchardt, Radosław Mądry, Monika Szarszewska, Katarzyna Gabalewicz, Dominika Pyszak, Petra Bretova, Beata Maćkowiak-Matejczyk, Wioletta Sawczuk, Monika Łączyńska-Madera, Dagmara Klasa-Mazurkiewicz, Angelika Gawlik-Urban, Magdalena Michalik, Zuzanna Borysiewicz, Ewa Iwańska, Mirosława Puskulluoglu, Paweł Blecharz, Renata Pacholczak-Madej

**Affiliations:** 1 Department of Gynecological Oncology, Krakow Branch, Maria Sklodowska-Curie National Research Institute of Oncology, Garncarska 11 Street, 31-115 Krakow, Poland; karolina.gorniak@krakow.nio.gov.pl (K.G.); angelika.gawlik10@gmail.com (A.G.-U.); ewa.iwanska@onet.pl (E.I.); pawel.blecharz@krakow.nio.gov.pl (P.B.); renata.pacholczak@uj.edu.pl (R.P.-M.); 2 Department of Proliferative Diseases, Comprehensive Oncology and Traumatology Center, Copernicus Memorial Hospital, 93-513 Lodz, Poland; m.habib@interia.pl; 3 Department of Radiotherapy and Gynaecological Oncology, Greater Poland Cancer Center in Poznan, 61-866 Poznań, Poland; ewa.burchardt@wco.pl; 4 Electroradiology Department, Poznan University of Medical Sciences, 61-701 Poznań, Poland; 5 Department of Oncology and Gynecological Oncology, Poznan University of Medical Sciences, 61-701 Poznań, Poland; r.madry.mac@gmail.com (R.M.); monika.szarszewska@gmail.com (M.S.); 6 Lower Silesian Oncology, Pulmonology and Hematology Center, 53-413 Wrocław, Poland; katarzyna.gabalewicz@dcopih.pl; 7 3rd Clinic of Radiotherapy and Chemotherapy, Gliwice Branch, Maria Sklodowska-Curie National Research Institute of Oncology, 00-001 Warszawa, Poland; d.pyszak@gmail.com; 8 Department of Obstetrics and Gynecology, Faculty of Medicine, Charles University in Hradec Kralove, 500 03 Hradec Králové, Czech Republic; bretova.petra@gmail.com; 9 Department of Obstetrics and Gynecology, University Hospital Hradec Kralove, 500 05 Nový Hradec Králové, Czech Republic; 10 Department of Gynaecological Oncology, Maria Sklodowska-Curie Bialystok Oncology Centre, 15-027 Białystok, Poland; bmackowiak@onkologia.bialystok.pl (B.M.-M.); wsawczuk@onkologia.bialystok.pl (W.S.); 11 Department of Gynecology, Gynecology Oncology and Obstetrics, Fryderyk Chopin University Hospital in Rzeszów, 35-055 Rzeszów, Poland; monikamadera1@gmail.com; 12 Department of Gynecology, Obstetrics, Gynecologic Oncology and Gynecologic Endocrinology, Medical University of Gdańsk, University Clinical Center, 80-952 Gdańsk, Poland; dklasa@gumed.edu.pl; 13 Clinical Oncology Department with Chemotherapy Subunit, Provincial Hospital Sain Luke in Tarnów, 33-100 Tarnów, Poland; 14 Faculty of Health Protection, Tarnów University, 33-100 Tarnów, Poland; 15 Department of Clinical Oncology, Prof. Kornel Gibiński University Clinical Center, Medical University of Silesia in Katowice, 40-055 Katowice, Poland; magda.michalikk@gmail.com; 16 Department of Oncology and Chemotherapy, Provincial Integrated Hospital in Elbląg, 82-300 Elbląg, Poland; zuza.borysiewicz@gmail.com; 17 Department of Clinical Oncology, Krakow Branch, Maria Sklodowska-Curie National Research Institute of Oncology, 00-001 Warszawa, Poland; mira.puskulluoglu@gmail.com; 18 Department of Anatomy, Jagiellonian University Medical College, 31-008 Kraków, Poland

**Keywords:** cervical cancer, immune checkpoint inhibitors, PD-1/PD-L1, cemiplimab, adverse events

## Abstract

Background: Immunotherapy has become an integral part of systemic treatment for cervical cancer (CC). This study assessed the safety profile of cemiplimab and the association between immune-related adverse events (irAEs) and treatment outcomes in patients with persistent, recurrent or metastatic CC. Methods: This ambispective, multicenter, real-world cohort study included 101 patients treated in 13 reference oncology centers as part of the PCCIG-01 study. We evaluated the frequency and severity of irAEs and their association with progression-free survival (PFS) and overall survival (OS). Survival outcomes were analyzed using the Kaplan–Meier method and Cox proportional hazards models, with *p* < 0.05 considered statistically significant. Results: After a median follow-up of 7.5 months, adverse events occurred in 45 patients (44.6%) and were mostly grade (G) 1–2. IrAEs were observed in 34 patients (33.7%). Endocrine toxicities predominated (*n* = 24, 58.5% of irAEs), followed by hepatic (*n* = 5, 12.2%) and gastrointestinal events (*n* = 4, 9.8%). G3 irAEs occurred in 8 patients (7.9%). Median PFS was 3.9 months (95% CI 2.9–5.6) in patients without irAEs and 10.9 months (95% CI 5.7–16.3) in those with irAEs (*p* = 0.03). Median OS was 15.3 months (95% CI 8.6–25.9) in patients without irAEs and was not reached in those with irAEs (95% CI 11.6-NR; *p* = 0.11). The development of irAEs was associated with a 54% reduction in the risk of progression (HR 0.46, 95% CI 0.27–0.80), with no statistically significant impact on OS. Conclusions: In exploratory analyses, the occurrence of irAEs was associated with improved PFS in cemiplimab-treated patients with persistent, recurrent or metastatic CC. Cemiplimab showed a manageable safety profile, with most toxicities being G1–G2.

## 1. Introduction

Cervical cancer (CC) remains a major global health burden and is currently the fourth most frequent malignancy worldwide, in terms of both incidence and mortality [1]. Even though the age-standardized mortality rate has decreased from 8.5 per 10,000 in 1990 to 7.1 per 100,000 in 2022, the absolute number of both new cases and deaths continues to rise globally, reflecting population growth and aging [1,2,3]. In Central and Eastern Europe, the burden of CC remains high. Compared with Western Europe, both the incidence and mortality remain significantly higher, with approximately 12–16 versus 6.8 new cases per 100,000 women and 6.1 versus 2.1 deaths per 100,000, respectively [4,5]. Reduced participation in preventive and screening programs contributes substantially to these disparities. Human papillomavirus (HPV) vaccination coverage in the target adolescent population reaches approximately 70–90% in Western and Northern European countries, whereas in Central and Eastern Europe it is considerably lower, at approximately 30–50%. In some settings, coverage is markedly below this range, as exemplified by the early phase of the Polish national HPV vaccination program initiated in 2023, where uptake has been reported at 8.67% [5,6]. Similarly, CC screening participation exceeds 70% in Western and Northern Europe, with the European Union average at 56%, but remains below 50% in parts of Central and Eastern Europe, contributing to delayed diagnosis and higher mortality [7,8,9].

While early-stage CC can often be cured with surgery and/or chemoradiotherapy (CRT), the prognosis for patients with recurrent, persistent or metastatic disease remains poor, with a 5-year survival rate of 17% [10,11]. For many years, platinum-based chemotherapy (CT), with or without the addition of anti-angiogenic therapy with bevacizumab, remained the standard treatment in advanced disease, providing only modest survival improvement [12]. In recent years, efforts to develop novel treatment options have led to the emergence of immune checkpoint inhibitors (ICIs) as a central part of CC management strategies, including both curative and palliative settings [13,14]. Cemiplimab (Libtayo^®^, Regeneron Pharmaceuticals, Tarrytown, NY, USA) is an anti-programmed cell death protein 1 (PD-1) ICI approved for the treatment of recurrent or metastatic cervical cancer after progression on prior platinum-based chemotherapy, irrespective of PD-L1 expression, based on the survival benefit demonstrated in the phase III EMPOWER-Cervical 1 trial [15].

In Poland, cemiplimab monotherapy has been reimbursed in second and further lines of treatment for patients with persistent, recurrent or metastatic CC previously treated with CT, since January 2025. Prior to reimbursement, access was limited to clinical trials or emergency access programs (EAP) conducted in specialized reference centers. In the Czech Republic, cemiplimab is available for CC patients exclusively through EAP.

As ICIs are increasingly incorporated into standard treatment across multiple malignancies, immune-related adverse events (irAEs) have become an important clinical issue. Beyond the need for early recognition and effective management to reduce morbidity and avoid treatment interruption, accumulating evidence suggests that the occurrence of irAEs may be associated with improved treatment outcomes and survival [16,17,18,19]. Although cemiplimab is an established treatment option in several malignancies, including cervical cancer, cutaneous squamous cell carcinoma, basal cell carcinoma and non-small cell lung cancer [20], real-world data on its safety profile remain limited. In our pilot studies, the development of irAEs was associated with significantly prolonged PFS [21], whereas thyroid irAEs appeared to be particularly strongly associated with both prolonged PFS and OS [22].

This study aimed to evaluate the association between the occurrence of irAEs and treatment outcomes in a larger real-world cohort of patients from Poland and the Czech Republic treated with cemiplimab to verify hypotheses from our pilot analysis. To our knowledge, this is the first comprehensive analysis of irAEs and their clinical relevance in patients with persistent, recurrent, or metastatic CC from Central and Eastern Europe.

## 2. Materials and Methods

### 2.1. Study Design

We conducted an ambispective, multicenter, real-world study in 12 reference oncology centers in Poland and 1 collaborating reference center in the Czech Republic within the Polish–Czech Cervical Cancer Immunotherapy Group (PCCIG)—project PCCIG-01 [23]. The patient cohort consisted of women with persistent, recurrent or metastatic cervical cancer previously treated with platinum-based CT with or without bevacizumab, who received at least one ICI treatment cycle between 18 October 2022 and 1 November 2025, with data cut-off on 15 February 2026. The study was conducted prospectively with regard to the timeline of cemiplimab administration, treatment response and adverse events (AEs) with their severity and management. Retrospective data acquisition included diagnosis, initial curative treatment, previous chemotherapeutic regimens, sites of metastatic spread (divided into 6 subgroups—nonregional lymph nodes, lungs, liver, bones, central nervous system [CNS] and other [including adrenal glands, kidneys, pancreas, pleura, mediastinum and muscles]), patient demographics and comorbidities (defined in File S1 of the Supplementary Materials). The study adhered to Strengthening the Reporting of Observational Studies in Epidemiology (STROBE) for cohort studies guidelines [24], with a detailed description of implemented strategies provided in File S2 of the Supplementary Materials.

### 2.2. Study Objectives

The primary objective was to evaluate the frequency and severity of irAEs in a real-world setting (RWS) of CC patients treated with cemiplimab. The secondary objective was to assess the impact of irAEs on survival parameters (overall survival [OS] and progression-free survival [PFS]) and treatment response (overall response rates [ORR] and disease control rates [DCR]). OS was defined as the time from initiation of cemiplimab treatment to death from any cause, including deaths unrelated to cancer or its treatment. PFS was defined as the time from initiation of cemiplimab treatment to documented disease progression or death from any cause, whichever occurred first. ORR was defined as complete remission (CR) or partial response (PR), whereas DCR was defined as CR, PR or stable disease (SD). Patients without an event were censored at the date of last follow-up.

Additionally, we aimed to contextualize our findings in relation to the pivotal trial [15] together with its subsequently published final analysis of overall survival [25] and other RWS. To support this narrative synthesis, we performed a structured search of the PubMed and Embase databases using the following keywords: [cervical cancer OR cervical carcinoma] AND [persistent OR recurrent OR metastatic OR advanced] AND [immunotherapy OR cemiplimab]. The search was performed between 1 March and 7 March 2026. No formal systematic review methodology or study selection process was performed.

### 2.3. Eligibility Criteria

Patients received cemiplimab within an EAP (Poland, Czech Republic) or according to the reimbursement criteria of the Polish Ministry of Health [26] (described in detail in File S3 of the Supplementary Materials), with eligibility criteria broadly consistent with those applied in the pivotal clinical trial [15]. The eligibility criteria included: squamous-cell carcinoma (SCC), adenocarcinoma (AC) or adenosquamous carcinoma (AC-SCC) of the cervix, irrespective of PD-L1 expression status, Eastern Cooperative Oncology Group (ECOG) performance status of 0–1, no symptomatic brain metastases, adequate renal, hepatic and bone marrow function, no uncontrolled concomitant malignancies, no autoimmune diseases (predefined exceptions—type 1 diabetes, treated autoimmune hypothyroidism, psoriasis, vitiligo) and measurable disease per Response Evaluation Criteria in Solid Tumors (RECIST) 1.1 criteria [27]. Within the EAP, ECOG performance status of 0–2 was accepted, more accurately reflecting the real-world clinical setting. Previous treatment with PD-1 or PD-L1 inhibitors was not permitted.

### 2.4. Intervention

Cemiplimab was administered in compliance with the approved dosing schedule specified in the summary of product characteristics [20]. The drug was delivered intravenously at a fixed dose of 350 mg every 3 weeks. Treatment was continued until radiologically or clinically confirmed PD, unacceptable toxicity or patient decision to discontinue treatment by withdrawing consent, whichever occurred first.

### 2.5. Follow-Up

Patients were monitored every 3 weeks with possible additional visits whenever necessary (e.g., AE management). Disease assessments were performed approximately every 12 weeks or earlier when justified (e.g., clinical suspicion of disease progression), using computed tomography (CT) of the chest, abdomen and pelvis, with additional body regions and imaging techniques (magnetic resonance imaging [MRI], positron emission tomography—computed tomography [PET-CT]), if clinically indicated. Radiological response was evaluated in accordance with RECIST version 1.1 criteria [27] to determine ORR and DCR. To ensure comprehensive OS follow-up, survival status was actively collected throughout the treatment and after its discontinuation until the end of the study. Data collection concluded on 15 February 2026. Patients were followed from the date of cohort entry until the end of the study period or death, whichever occurred first.

### 2.6. Evaluation of Adverse Events

All AEs arising during treatment and after discontinuation, until the end of the observational period, were collected and graded according to the Common Terminology Criteria for Adverse Events (CTCAE) version 5.0 [28]. They were categorized into 11 groups: endocrine, hepatic, pulmonary, cutaneous, gastrointestinal, rheumatologic, hematologic, neurologic, renal, ophthalmologic and general (including fatigue, infusion reactions and fever). Subsequently, AEs were classified as irAEs at each participating center by the treating investigators, if they were considered immune-mediated based on their clinical presentation, consistency with known immune-related toxicity profiles and absence of alternative causes. No centralized adjudication committee or inter-site harmonization procedure was implemented. All procedures for diagnosing and managing irAEs adhered to the European Society for Medical Oncology (ESMO) guidelines [29]. irAEs occurring after treatment discontinuation were included in the safety analyses. Time to onset was calculated from treatment initiation.

### 2.7. Ethical Considerations

The study protocol was approved by the Bioethics Committee of the Maria Sklodowska-Curie National Research Institute of Oncology in Poland (approval no. 93/2024, dated 21 November 2024). It was implemented uniformly across all participating centers in Poland and the Czech Republic. Standard institutional written informed consent was obtained from each patient prior to initiation of cemiplimab therapy. Given the partially retrospective design of the study and the use of anonymized electronic medical records, the requirement for additional, study-specific patient consent was waived by the Bioethics Committee.

### 2.8. Potential Sources of Bias and Strategies to Mitigate Them

Potential sources of bias were addressed as follows: selection bias was mitigated by applying uniform eligibility criteria across all participating centers and by enrolling patients consecutively. Information bias was reduced through standardized data collection procedures and radiologic response assessment according to RECIST version 1.1 [27]. To improve consistency in the recognition and management of irAEs, cases were evaluated in accordance with ESMO guidelines [29]. Potential confounding in survival analyses was addressed by adjusting multivariable models for prespecified, clinically relevant covariates. Data quality was ensured through systematic verification of extracted variables for completeness and consistency. Missing data were handled using a complete-case approach, and no imputation was performed.

### 2.9. Statistical Analyses

The study population comprised all consecutive patients treated with cemiplimab during the predefined study period; therefore, no formal sample size calculation was performed. The study population was stratified into two groups based on the occurrence of irAEs: patients who developed irAEs and those who did not. Categorical variables were presented as counts with percentages and compared using the χ^2^ test or Fisher’s exact test, as appropriate. Continuous variables were reported as medians with ranges and compared using the Wilcoxon rank-sum test. PFS and OS were estimated using the Kaplan–Meier method with median values and 95% confidence intervals (CIs) reported. Survival distributions were compared using the log-rank test. Associations between the occurrence of irAEs and outcomes were assessed using multivariable Cox proportional hazards models adjusted for prespecified clinically relevant covariates, including ECOG performance status and line of systemic therapy. The number of covariates was restricted in relation to the number of observed events; with two covariates included, the events-per-variable (EPV) was 33 for PFS and 20 for OS. The proportional hazards assumption was assessed using Schoenfeld residuals. All statistical tests were two-sided; *p*-value < 0.05 was considered statistically significant. All statistical analyses were performed using Stata version 19 (StataCorp LLC, College Station, TX, USA).

## 3. Results

### 3.1. Baseline Characteristics

A total of 101 patients were included in the study. The median age was 60 years (interquartile range [IQR] 46–68). Approximately two-thirds of patients were initially diagnosed with locally advanced CC (International Federation of Gynecology and Obstetrics [FIGO] stage III–IV), and most had previously received chemoradiotherapy (66.3%). At cemiplimab initiation, about two-thirds of the cohort had an ECOG performance status of 1. The most common metastatic sites were nonregional lymph nodes, lungs and liver. The most prevalent comorbidities were cardiovascular (30.7%) and renal (25.7%). Baseline demographic and clinical characteristics are summarized in Table 1 (with additional information provided inFile S4 of the Supplementary Materials). No statistically significant differences in baseline variables were observed; however, the absence of statistical significance does not imply equivalence.

### 3.2. Adverse Events Characteristics

AEs of any grade were observed in 45 of 101 patients (44.6%) with a total of 53 events recorded. The majority of affected patients experienced a single episode (*n* = 34, 75.6%), whereas 8 patients (17.8%) developed two events and 1 patient (2.2%) experienced three AEs. Most AEs were low-grade. Grade 1 toxicities accounted for 26 events (49.1%), grade 2 for 15 (28.3%), and grade 3 for 11 (20.8%). One grade 4 event (posterior reversible encephalopathy syndrome) was observed. No treatment-related deaths were reported.

**Figure 1:**
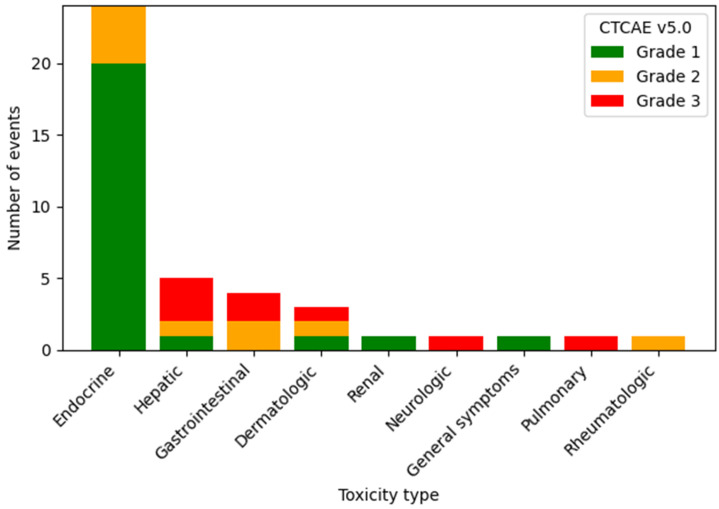
Distribution of immune-related adverse events by toxicity type and Common Terminology Criteria for Adverse Events CTCAE v.5.0.

IrAEs occurred in 34 patients (33.7% of the overall cohort) and accounted for 41 of all reported events. Endocrine toxicities (hypo-/hyperthyroidism) predominated, representing 24 events (58.5%), followed by hepatic (*n* = 5; 12.2%), gastrointestinal (*n* = 4; 9.8%) (3 cases of diarrhea/colitis, 1 case of gastritis) and dermatologic events (*n* = 3; 7.3%). Less common irAEs included single cases of renal, neurologic (encephalitis), pulmonary (interstitial pneumonitis) and rheumatologic events (arthralgia) as well as general symptoms (infusion reactions). The majority of irAEs were low grade, with grade 1 events accounting for 24 (58.5% of all irAEs) and grade 2 for 9 cases (22.0%), while grade 3 toxicities were reported in 8 cases (19.5%). No grade 4–5 irAEs were observed (Figure 1). Treatment discontinuation due to irAEs occurred in 6 patients (5.9%). In 2 patients (2.0%), grade 3 irAEs developed after treatment had already been discontinued due to disease progression; both were included in the safety analysis.

Non-immune AEs were observed in 11 patients (10.9% of the overall cohort). Of these, 10 were hematologic and consisted exclusively of anemia, while one case of grade 4 posterior reversible encephalopathy syndrome (PRES) was reported. The anemia cases were predominantly consistent with iron-deficiency anemia and/or anemia of chronic disease based on standard clinical and laboratory criteria. Hematologic adverse events were therefore classified as non-immune in origin, with no evidence suggestive of immune-mediated hemolytic anemia based on clinical assessment and evaluation of additional laboratory parameters, including lactate dehydrogenase (LDH) and bilirubin levels (not collected in the dataset). All reported AEs are summarized in Table 2.

The median time to the occurrence of irAEs was 2 months (IQR: 1–4 months), indicating that most immune-related toxicities developed early during treatment, prior to the first scheduled radiologic assessment at approximately 12 weeks. Systemic corticosteroids were required in 12 patients (35.3% of those experiencing irAEs) for the management of irAEs, without the need for additional immunosuppressive therapy.

### 3.3. Immune-Related Adverse Events and Survival Outcomes

After a median follow-up of 7.49 months (IQR: 4.37–12.22), patients received a median of 5 cemiplimab cycles (IQR 3–8), corresponding to a median treatment duration of 3.22 months (IQR 2.07–5.55). The ORR was 18.8% (19 patients), with responses observed in 11 patients without irAEs (16.4%) and 8 patients with irAEs (23.5%) (*p* = 0.39). Disease control was achieved in 43 patients (42.6%), including 24 patients without irAEs (35.8%) and 19 patients with irAEs (55.9%) (*p* = 0.052).

**Figure 2:**
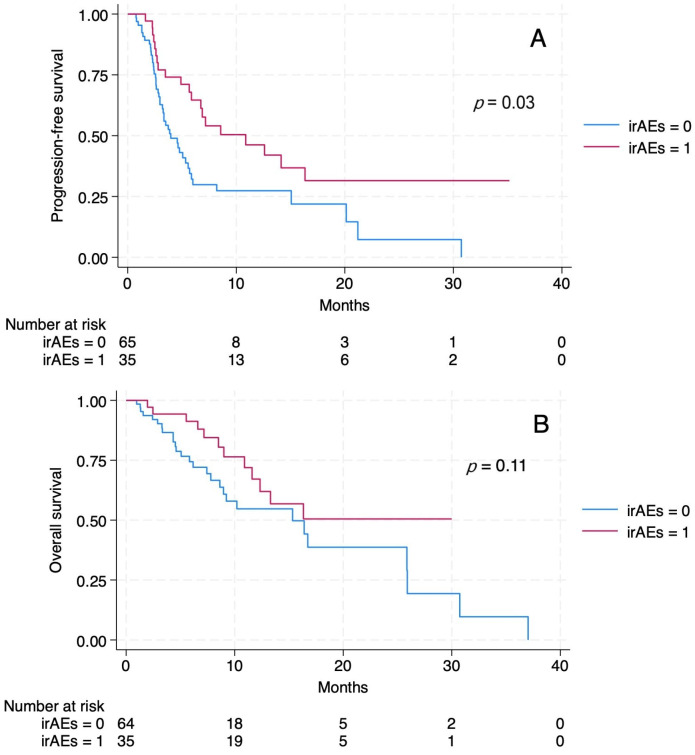
Kaplan–Meier curves for progression-free survival (**A**) and overall survival (**B**) depending on immune-related adverse events (irAEs).

PFS differed significantly according to the occurrence of irAEs. In patients without irAEs, PFS reached 3.97 months (95% CI 2.97–5.57) and 10.87 months (95% CI 5.67–16.33) in those who experienced irAEs (log-rank *p* = 0.03) (Figure 2A). In univariable Cox regression, the development of irAEs was associated with a reduced risk of progression or death (HR 0.49, 95% CI 0.29–0.84; *p* = 0.01) and this association remained significant after adjustment for predefined confounders (ECOG performance status and line of therapy: HR 0.46, 95% CI 0.27–0.80; *p* = 0.006). The proportional hazards assumption was met (Schoenfeld residuals global test *p* = 0.74).

For OS, a numerically longer OS was observed in patients with irAEs, although the difference did not reach statistical significance. In patients without irAEs, OS reached 15.33 months (95% CI 8.63–25.90) and was not reached (NR) in those with irAEs (95% CI 11.6–NR) (log-rank *p* = 0.11) (Figure 2B). In Cox regression, the occurrence of irAEs was not significantly associated with OS in univariable analysis (HR 0.58, 95% CI 0.29–1.14; *p* = 0.12) or after multivariable adjustment (HR 0.52, 95% CI 0.26–1.05; *p* = 0.07). The proportional hazards assumption was not violated (Schoenfeld residuals global test *p* = 0.99).

The treatment outcomes are summarized in Table 3.

### 3.4. Exploratory Subgroup Analyses

A subgroup analysis focusing on thyroid-related irAEs demonstrated a consistent association with improved outcomes. A significant association was observed for PFS, with longer PFS in patients experiencing thyroid irAEs (log-rank *p* = 0.008; HR 0.44, 95% CI 0.24–0.82), which remained significant in the multivariable model (HR 0.40, 95% CI 0.21–0.77; *p* = 0.005). Patients who developed thyroid irAEs showed a trend toward improved OS (log-rank *p* = 0.07; HR 0.45, 95% CI 0.19–1.08), which reached statistical significance after adjustment for predefined confounders (HR 0.40, 95% CI 0.17–0.98; *p* = 0.045).

PD-L1 CPS was available for 36 patients (35.6%), while the remaining 64.4% had an unknown status. Among evaluable patients, 26 (72.2%) were CPS-positive and 10 (27.8%) CPS-negative. No significant differences were observed for PFS (log-rank *p* = 0.57; CPS-positive vs. CPS-negative HR 0.70, 95% CI 0.29–1.69; *p* = 0.42; CPS-unknown vs. CPS-negative HR 0.65, 95% CI 0.29–1.46; *p* = 0.30). Similarly, no statistically significant differences in OS were observed between the groups (log-rank *p* = 0.80). For OS, the HR was 0.78 (95% CI 0.21–2.97; *p* = 0.72) for CPS-positive vs. CPS-negative patients and 0.68 (95% CI 0.20–2.30; *p* = 0.54) for CPS-unknown vs. CPS-negative patients. These results are presented in Figure S1 in the Supplementary Materials.

## 4. Discussion

This study provides the first real-world evidence from Central and Eastern Europe evaluating the safety profile of cemiplimab and the relationship between irAEs and treatment outcomes in patients with persistent, recurrent or metastatic CC. Overall, cemiplimab demonstrated a favorable and manageable safety profile, with most AEs being low-grade. Treatment discontinuation due to toxicity occurred in only a small proportion of patients. IrAEs were observed in approximately one-third of patients but were predominantly mild to moderate, with thyroid disorders representing the most frequent manifestation. Importantly, in exploratory analyses, the occurrence of irAEs was associated with significantly prolonged PFS and numerically longer OS, as well as a numerical increase in DCR. These findings should be interpreted cautiously given the observational design, limited follow-up and potential residual confounding.

The main strength of this study is its real-world, multicenter design, reflecting routine cemiplimab use across the PCCIG network. Importantly, the cohort consisted of patients who had not been previously exposed to ICIs, which is particularly relevant as immunotherapy is increasingly moving into earlier treatment settings in CC [13,30]. Thus, our population provides valuable evidence from an immunotherapy-naïve setting that may become less common in the future. Another strength is the regional relevance of the cohort, as Central and Eastern Europe continues to bear a high cervical cancer burden, yet it remains underrepresented in the RWS literature.

Interestingly, we found an association between the occurrence of irAEs and PFS without a statistically significant OS benefit. However, a trend toward improved OS was noted in patients experiencing irAEs (median OS NR vs. 15.33 months; multivariable Cox model *p* = 0.07), suggesting a potential association that did not reach statistical significance. This may reflect limited statistical power, particularly given the sample size and number of events. Secondly, OS is a multifactorial endpoint. It is influenced not only by the administered therapy, but also by other factors such as comorbidities, subsequent treatment lines and supportive care. In real-world clinical practice, additional variables, including delays in treatment initiation, therapy interruptions and access to comprehensive healthcare resources, may further influence survival. These factors may confound OS estimates in ways not typically observed in controlled clinical trials. Accordingly, PFS may more accurately capture the direct treatment effect of cemiplimab in this setting.

In the present study, thyroid-related irAEs were associated with improved outcomes, particularly for PFS, where the effect remained significant after adjustment for key clinical variables. For OS, a non-significant trend in univariable analysis became significant in the multivariable model, likely reflecting the influence of baseline confounding factors. These findings are consistent with our pilot study [22] and support the hypothesis that endocrine irAEs, particularly thyroid dysfunction, may reflect enhanced immune activation. The potential biological mechanisms underlying this association have been discussed in detail in our previous report [22]. However, given the observational nature of the study and the limited number of events, these results should be interpreted with caution and considered hypothesis-generating.

The association between irAEs and improved treatment outcomes, including ORR, PFS and OS, has been demonstrated in several systematic reviews and meta-analyses [31,32,33]. ICIs stimulate anti-tumor immunity by inhibiting negative regulatory pathways restraining T-cell activation and thereby restore and strengthen the cytotoxic T-cell response against tumor cells [34]. However, this non-specific mechanism may target not only tumor cells but healthy tissues as well, resulting in autoimmune-like toxicities [33,34]. Consequently, the occurrence of irAEs may serve as an indicator of immune system activation and enhanced anti-tumor immune response [32,33,34,35,36]. This association appears to be particularly strong for endocrine and dermatologic toxicities in patients treated with anti-PD-1/PD-L1 inhibitors [31,33]. Importantly, evidence suggests that mild to moderate irAEs (grade 1–2) are most strongly associated with improved survival outcomes, while more severe toxicities lead to treatment discontinuation and shortened OS [33,37]. These findings are consistent with the present study, in which the majority of irAEs were mild to moderate. In our cohort, the median interval between initiation of cemiplimab therapy and onset of irAEs was 2 months. This observation underscores the importance of early and systematic monitoring and follow-up, as timely recognition and prompt management of irAEs may prevent progression to high-grade toxicity and facilitate continuation of treatment or subsequent rechallenge. Notably, among patients who developed irAEs requiring immunosuppressive intervention, systemic corticosteroids alone were sufficient in all cases, without additional immunosuppressive therapy.

Given the relatively low incidence of CC in high-income countries and the restricted availability of immunotherapy in low- and middle-income countries—where the disease burden is greatest—real-world data on safety, toxicity and clinical outcomes of ICI-based treatments remains limited. This paucity of data highlights the scientific and clinical relevance of the present research project. The findings of the conducted narrative review of the literature are summarized in Table 4. Additionally, a direct comparison between our real-world cohort’s and the pivotal trial’s [15] safety and efficacy outcomes is provided in Table 5. Differences in AE reporting between clinical trials and real-world settings may partly explain the lower incidence observed in our cohort. Concerning treatment outcomes, the identified studies report a median PFS ranging from 2.8 to 5.7 months and a median OS between 8.4 and 12.0 months [15,21,25,38,39]. The study by Kitami et al. [40] was an exception, reporting a PFS and OS of 10.4 and 22 months, respectively. However, direct comparison with this cohort is limited, as the population differed substantially from those included in other reports—the study permitted the use of combined chemo-immunotherapy and allowed ICI rechallenge, both of which may have influenced the results. In our study the median PFS and OS were 5.6 and 16.3 months, respectively, which appear consistent with previously reported outcomes. Similarly, the ORR reported in the literature ranged from 16.4% to 23% [15,21,25,38], while in our cohort it reached 18.8%, further supporting the comparability of treatment efficacy across studies. In the subgroup analysis of patients treated with cemiplimab monotherapy in the study by Kitami et al. [40], the ORR reached only 5.6%. Notably, 44.4% of patients who received cemiplimab in this trial were treated in the setting of ICI rechallenge, which further limits direct comparison with other studies.

In terms of safety, irAEs were reported in approximately 15.7–45.0% of patients in previous studies, with grade ≥ 3 irAEs occurring in 3.4–19.0% of cases [15,22,38,39,40]. In our cohort, the incidence of irAEs (33.7%) and grade ≥ 3 irAEs (7.9%) was comparable to previously published data. Several factors may account for the variability in reported toxicity rates, including differences between clinical trial populations and real-world cohorts, more comprehensive reporting of irAEs in observational studies and potential heterogeneity in patient characteristics or prior treatments. Interpretation is further hindered by variability in methodological approaches, as some analyses reported overall AEs or treatment-emergent AEs rather than specifically irAEs, and the proportion of high-grade irAEs was not consistently reported as a distinct outcome. In line with the findings reported by Tuninetti et al. [39] and with our previously published pilot analyses [21,22], the occurrence of irAEs was associated with improved PFS and a trend toward improved OS and DCR. However, this association has been inconsistently reported across studies.

Several limitations of this study should be acknowledged. First, the follow-up duration was relatively short, limiting the maturity and robustness of survival analyses; however, at the time of data cut-off, only 22.8% of patients (*n* = 23) remained on treatment. Second, the study may have been subject to selection bias, as the EAPs and reimbursement programs are implemented predominantly in specialized referral institutions. This issue may be further reinforced by the strictly regulated eligibility criteria of the reimbursement program. Third, our cohort was ethnically homogeneous and consisted exclusively of Caucasian patients from Central and Eastern Europe. Given potential genetic, environmental and healthcare system differences, caution is warranted when extrapolating these findings to other populations and further studies in more diverse cohorts are needed. Fourth, reporting bias cannot be excluded. Certain irAEs, particularly mild or nonspecific symptoms such as fatigue, may have been underrecognized, potentially affecting the reported frequency and severity of toxicities. irAEs attribution was performed locally at each participating center without centralized adjudication, which may have introduced inter-observer variability and potential misclassification bias despite the use of standard clinical criteria. Another limitation relates to the analysis of the incidence of irAEs as a binary variable. Because patients must remain on treatment long enough for an irAE to develop, the observed association between irAEs and improved outcomes may have been influenced by immortal time bias. Therefore, this relationship should be interpreted with caution. Given the early onset of most irAEs and the limited number of patients not reaching 3 months of follow-up (*n* = 16), additional landmark analyses were considered unlikely to provide robust incremental information in this cohort. Finally, treatment response was assessed locally at participating centers and no central radiology review was performed. Although response evaluation was based on RECIST version 1.1, inter-site variability in imaging interpretation cannot be excluded.

## 5. Conclusions

In this real-world cohort, the occurrence of irAEs was associated with longer PFS in patients with persistent, recurrent, or metastatic CC treated with cemiplimab. Cemiplimab demonstrated a manageable safety profile with most irAEs being grade 1–2. These findings are exploratory and hypothesis-generating, and further studies in larger and more diverse populations are needed to confirm these observations.

## Figures and Tables

**Table 1:** Baseline characteristics of the enrolled patients (*n* = 101).

		All (*n* = 101)	irAEs (*n* = 34)	no irAEs (*n* = 67)	*p* Value
Age–median (IQR)		60 (46–68)	59 (49–66)	60 (45–69)	0.88
ECOG performance status at cemiplimab initiation—*n* (%)	0	17 (16.8)	8 (23.5)	9 (13.4)	0.26
	1	70 (69.3)	20 (58.8)	50 (74.6)	0.11
	2	14 (13.9)	6 (17.7)	8 (12.0)	0.51
Histological subtype—*n* (%)	Squamous cell carcinoma	66 (65.3)	24 (70.6)	42 (62.7)	0.52
	Adenocarcinoma	34 (33.7)	10 (29.4)	24 (35.8)	0.60
	Adenosquamous carcinoma	1 (1.0)	0 (0)	1 (1.5)	1.0
FIGO stage at primary diagnosis—*n* (%)	I	14 (13.9)	2 (5.9)	12 (17.9)	0.14
	II	18 (17.8)	8 (23.5)	10 (14.9)	0.34
	III	34 (33.7)	10 (29.4)	24 (35.9)	0.61
	IV	35 (34.6)	14 (41.2)	21 (31.3)	0.36
Grade—*n* (%)	1	8 (7.9)	4 (11.8)	4 (6.0)	0.45
	2	50 (49.5)	18 (52.9)	32 (47.7)	0.65
	3	15 (14.9)	2 (5.9)	13 (19.4)	0.08
	Unknown	28 (27.7)	10 (29.4)	18 (26.9)	1.0
Positive PD-L1 CPS (≥1)—*n* (%)	Yes	26 (25.7)	8 (23.5)	18 (26.9)	1.0
	No	10 (9.9)	3 (8.8)	7 (10.4)	1.0
	Unknown	65 (64.4)	23 (67.7)	42 (62.7)	0.78
HPV status—*n* (%)	Positive	13 (12.9)	4 (11.8)	9 (13.4)	1.0
	Negative	6 (5.9)	3 (8.8)	3 (4.5)	0.65
	Unknown	82 (81.2)	27 (79.4)	55 (82.1)	1.0
Primary radical surgery—*n* (%)	Yes	28 (27.7)	9 (26.5)	19 (28.4)	1.0
	No	73 (72.3)	25 (73.5)	48 (71.6)	1.0
Chemoradiotherapy—*n* (%)	Yes	67 (66.3)	23 (67.6)	44 (65.7)	1.0
	No	29 (28.7)	10 (29.4)	19 (28.3)	1.0
	Radiotherapy alone	5 (5.0)	1 (3.0)	4 (6.0)	0.66
Brachytherapy	Yes	59 (58.4)	18 (52.9)	41 (61.2)	0.46
	No	39 (38.6)	16 (47.1)	23 (34.3)	0.22
	Unknown	3 (3.0)	0 (0)	3 (4.5)	0.56
Number of previous lines of CT—*n* (%)	1	68 (67.3)	21 (61.7)	47 (70.1)	0.47
	2	27 (26.7)	12 (35.3)	15 (22.4)	0.19
	3	5 (5.0)	0 (0)	5 (7.5)	0.18
	4	1 (1.0)	1 (3.0)	0 (0)	0.36
Previous bevacizumab treatment—*n* (%)	Yes	54 (53.5)	18 (52.9)	36 (53.7)	1.0
	No	47 (46.5)	16 (47.1)	31 (46.3)	1.0

Abbreviations: CPS—combined positive score; CT—chemotherapy; ECOG—Eastern Cooperative Oncology Group; FIGO—International Federation of Gynecology and Obstetrics; HPV—human papillomavirus; IQR—interquartile range; irAEs—immune-related adverse events; PD-L1—programmed death-ligand 1.

**Table 2:** Categories of reported adverse events.

Category	Grade 1	Grade 2	Grade 3	Grade 4	Total n (%)
**Immune-Related AEs**					
Endocrine	20	4	0	0	24 (45.3%)
Hepatic	1	1	3	0	5 (9.4%)
Gastrointestinal	0	2	2	0	4 (7.5%)
Dermatologic	1	1	1	0	3 (5.7%)
Renal	1	0	0	0	1 (1.9%)
Neurologic	0	0	1	0	1 (1.9%)
Pulmonary	0	0	1	0	1 (1.9%)
Rheumatologic	0	1	0	0	1 (1.9%)
General symptoms	1	0	0	0	1 (1.9%)
**Subtotal irAEs**	**24**	**9**	**8**	**0**	**41 (77.4%)**
**Non-immune AEs**					
Hematologic (anemia *)	3	5	3	0	11 (20.8%)
PRES	0	0	0	1	1 (1.9%)
**Subtotal non-irAEs**	**3**	**5**	**3**	**1**	**12 (22.6%)**
**Total AEs**	**27**	**14**	**11**	**1**	**53 (100%)**

* Predominantly iron-deficiency anemia and/or anemia of chronic disease, without evidence of immune-mediated etiology. *Abbreviations: AEs—adverse events; irAEs—immune-related adverse events; PRES—posterior reversible encephalopathy syndrome.*

**Table 3:** Treatment outcomes.

	All (*n* = 101)	irAEs (*n* = 34)	No irAEs (*n* = 67)	*p* Value
DCR—*n* (%)	43 (42.6)	19 (55.9)	24 (35.8)	0.052
ORR—*n* (%)	19 (18.8)	8 (23.5)	11 (16.4)	0.39
PFS—median, months (95% CI)	5.6 (3.9–7.2)	10.9 (5.7–16.3)	3.9 (2.9–5.6)	0.03 *
OS—median, months (95% CI)	16.3 (10.9–25.9)	NR (11.6–NR)	15.3 (8.6–25.9)	0.11

*—statistical significance—*p* < 0.05. Abbreviations: CI—confidence interval; DCR—disease control rate; irAEs—immune-related adverse events; NR—not reached; ORR—objective response rate; OS—overall survival; PFS—progression-free survival.

**Table 4:** Summary of relevant literature.

Ref.	Study Design	Population	Treatment	Outcomes	Safety
Tewari et al. [15]	EMPOWER-Cervical 1—open-label multicenter randomized phase 3 trial	608 patients with recurrent or metastatic CC with disease progression after first-line platinum-based CT, irrespective of PD-L1 expression status; ECOG 0–1	Cemiplimab vs. investigator’s choice single-agent chemotherapy (1:1 randomization)	Median follow-up 18.2 months; median OS: 12.0 months (cemiplimab) vs. 8.5 months (CT) (HR 0.69; 95% CI 0.56–0.84; *p* < 0.001); median PFS: 2.8 months (cemiplimab) vs. 2.9 months (CT) (HR 0.75, 95% CI 0.63–0.89, *p* < 0.001)	AEs—88.3% (cemiplimab) vs. 91.4% (CT); irAEs (cemiplimab)—15.7%; grade ≥ 3 irAEs (cemiplimab)—5.3%
Oaknin et al. [25]	EMPOWER-Cervical 1—open-label multicenter randomized phase 3 trial—final OS analysis	As above	As above	Median follow-up 47.3 months; median OS: 11.7 months (cemiplimab) vs. 8.5 months (CT) (HR 0.67, 95% CI: 0.56–0.80, *p* < 0.00001)	AEs: 89.7% (cemiplimab) vs. 91.7% (CT); TRAEs: 57.3% (cemiplimab) vs. 81.7% (CT); irAEs frequency and severity not reported
Hasegawa et al. [38]	Post hoc subgroup analysis of phase 3 EMPOWER-Cervical 1 trial, conducted in Japan	56 patients with recurrent or metastatic CC with disease progression after first-line platinum-based CT, irrespective of PD-L1 expression status; ECOG 0–1	As above	Median follow-up 13.6 months; median OS: 8.4 months (cemiplimab) vs. 9.4 months (CT) (HR 0.86; 95% CI 0.43–1.68); median PFS: 4.0 months (cemiplimab) vs. 3.7 months (CT) (HR 0.90; 95% CI 0.50–1.61)	TEAEs: 79.3% (cemiplimab) vs. 100% (CT); grade ≥ 3 AEs: 37.9% (cemiplimab) vs. 66.7% (CT); irAEs (cemiplimab): 24.1%; grade ≥ 3 irAEs (cemiplimab)—3.4%
Tuninetti et al. [39]	Real-world multicenter retrospective cohort study MITO 44	128 patients with recurrent, persistent or metastatic CC previously treated with platinum-based CT, irrespective of PD-L1 expression status; ECOG 0–2	Cemiplimab	Median OS: 12.0 months; median PFS: 4.0 months; similar PFS and OS in patients with ECOG PS of 0, 1 and 2; irAEs statistically significant for PFS and OS	irAEs—18.0%; grade ≥ 3 irAEs—not reported
Kitami et al. [40]	Multicenter observational study	100 patients with recurrent, persistent or advanced CC, irrespective of PD-L1 expression status; ECOG 0–3; ICI rechallenge allowed	Pembrolizumab +/− CT +/− bevacizumab, or cemiplimab monotherapy	All patients: ORR—62%; DCR—77%; median PFS—10.4 months; median OS—22 months Cemiplimab subgroup (18 patients [18%]): ORR—5.6%	All patients: irAEs—45.0% grade ≥ 3 irAEs—19.0%; treatment-related deaths—6% Cemiplimab subgroup—not reported
Pacholczak-Madej et al. [21]	Real-world multicenter retrospective study	39 patients with recurrent/metastatic CC, irrespective of PD-L1 expression status; ECOG 0–2	Cemiplimab (37 patients) or pembrolizumab (2 patients)	Median follow-up—8.8 months; median PFS—5.7 months; median OS—10.9 months; prolonged PFS in patients with irAEs (HR 0.2, 95% CI: 0.09–0.6)	AEs—59% of patients; grade ≥ 3 AEs—14.8%
Pacholczak-Madej et al. [22]	Real-world multicenter ambispective observational study	37 patients with recurrent/metastatic CC, irrespective of PD-L1 expression status; ECOG 0–2	Cemiplimab	Median follow-up—9.2 months; significantly longer PFS (HR 0.2, 95% CI 0.07–0.6; *p* = 0.004) and OS (HR 0.2; 95% CI 0.05–0.9; *p* = 0.04) in patients with thyroid irAEs	irAEs—40.5%; most common irAEs—thyroid dysfunction (64.7% of irAEs)

Abbreviations: AE—adverse event; CC—cervical cancer; CI—confidence interval; CT—chemotherapy; DCR—disease control rate; HR—hazard ratio; irAE—immune-related adverse event; NR—not reached; ORR—objective response rate; OS—overall survival; PD-L1—programmed death-ligand 1; PFS—progression-free survival; PS—performance status; TEAE—treatment-emergent adverse event.

**Table 5:** Comparison of efficacy and safety between PCCIG cohort and EMPOWER-Cervical 1 cohort.

	PCCIG	EMPOWER-Cervical 1
Number of participants	101	304
ECOG performance status	0–2	0–1
Median follow-up	7.5 months	18.2 months
Median PFS	5.6 months	2.8 months
Median OS	16.3 months	12.0 months
ORR	18.8%	16.4%
DCR	42.6%	57.6%
AEs	44.6%	88.3%
Grade ≥ 3 AEs	11.9%	45.0%
AEs leading to death	0	1.7%
irAEs	33.7%	15.7%
Grade ≥ 3 irAEs	7.9%	5.3%

Abbreviations: AEs—adverse events; DCR—disease control rate; ECOG—Eastern Cooperative Oncology Group; irAEs—immune-related adverse events; ORR—objective response rate; OS—overall survival; PFS—progression-free survival.

## Data Availability

The raw data supporting the conclusions of this article will be made available by the authors on request.

## Supplementary Materials

The following supporting information can be downloaded at: https://www.mdpi.com/article/10.3390/antib15030042/s1, File S1: Definitions of comorbidities [41]; File S2: STROBE Statement—checklist of items that should be included in reports of cohort studies; File S3: National reimbursement criteria for cemiplimab in cervical cancer in Poland; File S4: Baseline characteristics of the enrolled patients (*n* = 101)—additional information; Figure S1: Kaplan–Meier curves for A—overall survival (OS) and B—progression-free survival (PFS) stratified by programmed death-ligand 1 (PD-L1) combined positive score (CPS) status.

## References

[1] Ferlay J., Ervik M., Lam F., Laversanne M., Colombet M., Mery L., Piñeros M., Znaor A., Soerjomataram I., Bray F. Global Cancer Observatory: Cancer Today. Lyon, France: International Agency for Research on Cancer. 2024.

[2] Azangou-Khyavy M., Ghasemi E., Rezaei N., Khanali J., Kolahi A.A., Malekpour M.R., Heidari-Foroozan M., Nasserinejad M., Mohammadi E., Abbasi-Kangevari M. (2024). Global, regional, and national quality of care index of cervical and ovarian cancer: A systematic analysis for the global burden of disease study 1990–2019. BMC Womens Health.

[3] Yi M., Li T., Niu M., Luo S., Chu Q., Wu K. (2021). Epidemiological trends of women’s cancers from 1990 to 2019 at the global, regional, and national levels: A population-based study. Biomark. Res..

[4] Wojtyla C., Ciebiera M., Kowalczyk D., Panek G. (2020). Cervical Cancer Mortality in East-Central European Countries. Int. J. Environ. Res. Public Health.

[5] Bencina G., Sabale U., Morais E., Ovcinnikova O., Oliver E., Shoel H., Meiwald A., Hughes R., Weston G., Sundström K. (2024). Burden and indirect cost of vaccine-preventable cancer mortality in Europe. J. Med. Econ..

[6] Poniewierza P., Śniadecki M., Musielak O., Raza A., Safari Y., Piątek-Dalewska O., Danielkiewicz M., Mazur A., Amerek Z., Khan S.R. (2015). Initial Coverage and Regional Disparities of the National HPV Vaccination Program in Poland: A Cross-Sectional Analysis. Healthcare.

[7] Organisation for Economic Co-Operation and Development (2024). Health at a Glance: Europe 2024.

[8] OECD (2024). Beating Cancer Inequalities in the EU.

[9] OECD (2026). Delivering High Value Cancer Care.

[10] Cohen P.A., Jhingran A., Oaknin A., Denny L. (2019). Cervical cancer. Lancet.

[11] Galicia-Carmona T., Arango-Bravo E.A., Coronel-Martínez J.A., Cetina-Pérez L., Vanoye-Carlo E.G., Villalobos-Valencia R., García-Pacheco J.A., Cortés-Esteban P. (2024). Advanced, recurrent, and persistent cervical cancer management: In the era of immunotherapy. Front. Oncol..

[12] Tewari K.S., Sill M.W., Long H.J., Penson R.T., Huang H., Ramondetta L.M., Landrum L.M., Oaknin A., Reid T.J., Leitao M.M. (2014). Improved Survival with Bevacizumab in Advanced Cervical Cancer. N. Engl. J. Med..

[13] Ogasawara A., Hasegawa K. (2025). Recent advances in immunotherapy for cervical cancer. Int. J. Clin. Oncol..

[14] Nasso C., Puglisi S., Rebuzzi S.E., Errigo V., Rosa F., Chiola I., Lazzari C., Musizzano Y., Venturino E., Gastaldo A. (2025). Immune checkpoint inhibitors in gynecological cancers: A narrative review on the practice-changing trials. Immunotherapy.

[15] Tewari K.S., Monk B.J., Vergote I., Miller A., de Melo A.C., Kim H.-S., Kim Y.M., Lisyanskaya A., Samouëlian V., Lorusso D. (2022). Survival with Cemiplimab in Recurrent Cervical Cancer. N. Engl. J. Med..

[16] Monaca F., Gomez-Randulfe I., Parreira A.S., Longo V., Galetta D., Pilotto S., Polidori S., Cantale O., Stefani A., Vita E. (2025). Correlation between irAEs and survival outcomes in patients with ES-SCLC treated with first-line chemoimmunotherapy. Eur. J. Cancer.

[17] Yan W., Qin L., Han Y., Jia X., Wu J. (2025). Impact of Immune-Related Adverse Events on Survival in Patients With Gastrointestinal Cancer Treated With Immune Checkpoint Inhibitors: A Meta-Analysis. Clin. Transl. Gastroenterol..

[18] Indini A., Di Guardo L., Cimminiello C., Prisciandaro M., Randon G., De Braud F., Del Vecchio M. (2019). Immune-related adverse events correlate with improved survival in patients undergoing anti-PD1 immunotherapy for metastatic melanoma. J. Cancer Res. Clin. Oncol..

[19] Yamamoto K., Hirano H., Hirose T., Shoji H., Okita N., Takashima A., Kato K. (2025). Association Between Immune-Related Adverse Events and Treatment Outcomes in Advanced Gastric Cancer Patients Receiving Nivolumab Plus Chemotherapy: A Retrospective Study. Cancer Med..

[20] Summary of Product Characteristics Libtayo (Cemiplimab). https://www.ema.europa.eu/en/documents/product-information/libtayo-epar-product-information_en.pdf.

[21] Pacholczak-Madej R., Lisik-Habib M., Mądry R., Szarszewska M., Borysiewicz Z., Gabalewicz K., Iwańska E., Szatkowski W., Puskulluoglu M., Jakubowicz J. (2025). Real-world outcomes of immune checkpoint inhibitor monotherapy in later lines of recurrent/metastatic cervical cancer—Evidence from a rescue access program in Poland. Wspolczesna Onkol. Oncol..

[22] Pacholczak-Madej R., Lisik-Habib M., Mądry R., Szarszewska M., Borysiewicz Z., Gabalewicz K., Iwańska E., Szatkowski W., Puskulluoglu M., Jakubowicz J. (2025). Thyroid disorders as predictors of cemiplimab efficacy in recurrent/metastatic cervical cancer: Real-world evidence from Poland. Front. Immunol..

[23] OSF PCCIG Project: Real-World Outcomes of Cemiplimab in Recurrent/Metastatic Cervical Cancer—A Polish–Czech Multicenter Collaboration. https://osf.io/zpn9j/wiki?wiki=zdpkf.

[24] Checklists—STROBE. https://www.strobe-statement.org/checklists/.

[25] Oaknin A., Monk B.J., de Melo A.C., Kim H.S., Kim Y.M., Lisyanskaya A.S., Samouëlian V., Lorusso D., Damian F., Chang C.-L. (2025). Cemiplimab in recurrent cervical cancer: Final analysis of overall survival in the phase III EMPOWER-Cervical 1/GOG-3016/ENGOT-cx9 trial. Eur. J. Cancer.

[26] Program Lekowy Ministerstwa Zdrowia. https://www.gov.pl/web/zdrowie/choroby-onkologiczne.

[27] Eisenhauer E.A., Therasse P., Bogaerts J., Schwartz L.H., Sargent D., Ford R., Dancey J., Arbuck S., Gwyther S., Mooney M. (2009). New response evaluation criteria in solid tumours: Revised RECIST guideline (version 1.1). Eur. J. Cancer.

[28] National Cancer Institute (2017). Common Terminology Criteria for Adverse Events (CTCAE) v5.0. https://www.meddra.org/.

[29] Haanen J., Obeid M., Spain L., Carbonnel F., Wang Y., Robert C., Lyon A., Wick W., Kostine M., Peters S. (2022). Management of toxicities from immunotherapy: ESMO Clinical Practice Guideline for diagnosis, treatment and follow-up. Ann. Oncol..

[30] Cibula D., Raspollini M.R., Planchamp F., Centeno C., Chargari C., Felix A., Fischerová D., Jahnn-Kuch D., Joly F., Kohler C. (2023). ESGO/ESTRO/ESP Guidelines for the management of patients with cervical cancer—Update 2023. Int. J. Gynecol. Cancer.

[31] Zhou X., Yao Z., Yang H., Liang N., Zhang X., Zhang F. (2020). Are immune-related adverse events associated with the efficacy of immune checkpoint inhibitors in patients with cancer? A systematic review and meta-analysis. BMC Med..

[32] Das S., Johnson D.B. (2019). Immune-related adverse events and anti-tumor efficacy of immune checkpoint inhibitors. J. Immunother. Cancer.

[33] Hussaini S., Chehade R., Boldt R.G., Raphael J., Blanchette P., Maleki Vareki S., Fernandes R. (2021). Association between immune-related side effects and efficacy and benefit of immune checkpoint inhibitors—A systematic review and meta-analysis. Cancer Treat. Rev..

[34] Postow M.A., Sidlow R., Hellmann M.D. (2018). Immune-Related Adverse Events Associated with Immune Checkpoint Blockade. N. Engl. J. Med..

[35] Pacholczak-Madej R., Drobniak A., Stokłosa Ł., Bidas A., Dobrzańska J., Grela-Wojewoda A., Roman A., Tusień-Małecka D., Walocha J., Blecharz P. (2024). Adverse events after nivolumab and ipilimumab combined immunotherapy in advanced renal cell carcinoma: A multicentre experience in Poland. BMC Cancer.

[36] Pacholczak-Madej R., Grela-Wojewoda A., Puskulluoglu M., Lompart J., Las-Jankowska M., Krawczak K., Wrona E., Zaręba L., Żubrowska J., Walocha J. (2022). Early Effects of Nivolumab and Ipilimumab Combined Immunotherapy in the Treatment of Metastatic Melanoma in Poland: A Multicenter Experience. Biomedicines.

[37] Wang Y., Zhou S., Yang F., Qi X., Wang X., Guan X., Shen C., Duma N., Vera Aguilera J., Chintakuntlawar A. (2019). Treatment-Related Adverse Events of PD-1 and PD-L1 Inhibitors in Clinical Trials: A Systematic Review and Meta-analysis. JAMA Oncol..

[38] Hasegawa K., Takahashi S., Ushijima K., Okadome M., Yonemori K., Yokota H., Vergote I., Monk B.J., Tewari K.S., Fujiwara K. (2024). Cemiplimab monotherapy in Japanese patients with recurrent or metastatic cervical cancer. Cancer Med..

[39] Tuninetti V., Virano E., Salutari V., Ricotti A., Pisano C., Ducceschi M., Turitto G., Scandurra G., Petrella M.C., Forestieri V. (2024). Real-life efficacy and safety of cemiplimab in advanced cervical cancer from a nominal use program in Italy: The MITO 44 study. Eur. J. Cancer.

[40] Kitami K., Kuji S., Kamiya N., Machida H., Koike J., Watanabe R., Hirasawa T., Suzuki N., Miyagi E., Kato K. (2026). Real-world safety and efficacy of immune checkpoint inhibitors in Japanese patients with persistent, recurrent, or metastatic cervical cancer: A multicenter prospective and retrospective study. Int. J. Clin. Oncol..

[41] Loscalzo J., Fauci A., Kasper D., Hauser S., Longo D., Jameson J. (2022). Harrison’s Principles of Internal Medicine, Twenty-First Edition (Vol.1 & Vol.2).

