# Supplementary material for: Immune-Related Adverse Events of Cemiplimab Therapy in Advanced Cervical Cancer—Data from the Polish–Czech Cervical Cancer Immunotherapy Group (PCCIG-01) with a Review of the Literature
Source: Antibodies (Basel). 2026 May 18;15(3):42. doi: 10.3390/antib15030042 (PMC13214483; doi:10.3390/antib15030042)
Supplement: Supplementary file 1 [file antibodies-15-00042-s001.zip › antibodies-4250997-supplementary.pdf]

Supplementary Materials

File S1. Definitions of comorbidities [1]

- **obesity -** body mass index (BMI) ≥30 kg/m², where BMI is calculated as body weight in kilograms divided by the square of height in meters (kg/m²)
- **cardiovascular**– history of cardiovascular disorders, including coronary artery disease, heart failure, arrhythmias, valvular heart disease or arterial hypertension requiring pharmacologic treatment
- **pulmonary**– chronic respiratory disorders such as chronic obstructive pulmonary disease (COPD), asthma, interstitial lung disease, pulmonary hypertension or other lung diseases requiring ongoing medical management
- **nephrological**– chronic kidney disease defined as structural or functional kidney abnormalities persisting ≥ 3 months or estimated glomerular filtration rate (eGFR) < 60 mL/min/1.73 m²
- **endocrinological**– chronic endocrine disorders including type 2 diabetes mellitus, thyroid disease (hypothyroidism or hyperthyroidism), adrenal disorders, pituitary disorders or other clinically significant endocrine diseases
- **autoimmune diseases** – systemic or organ-specific chronic disorders characterized by immune-mediated damage to self-tissues - in general, a contraindication to cemiplimab treatment with 4 acceptable exceptions: type 1 diabetes, treated autoimmune hypothyroidism, psoriasis, vitiligo
- **deep vein thrombosis** – presence of thrombus in the deep venous system, including pulmonary embolism, confirmed by imaging studies (e.g., Doppler ultrasound, CT venography) or documented in medical records
- **hypercholesterolemia** – total serum cholesterol ≥ 200 mg/dL (≥ 5.2 mmol/L) or the use of lipid-lowering therapy

File S2. STROBE Statement [2]— checklist of items that should be included in reports of *cohort studies* in relation to our study.

	Item No	Recommendation	Page No
**Title and abstract**	1	(*a*) Indicate the study's design with a commonly used term in the title or the abstract	1
		(*b*) Provide in the abstract an informative and balanced summary of what was done and what was found	3
**Introduction**			
Background/rationale	2	Explain the scientific background and rationale for the investigation being reported	4-5
Objectives	3	State specific objectives, including any prespecified hypotheses	4-5
**Methods**			
Study design	4	Present key elements of study design early in the paper	5-6
Setting	5	Describe the setting, locations, and relevant dates, including periods of recruitment, exposure, follow-up, and data collection	6-7
Participants	6	(*a*) Give the eligibility criteria, and the sources and methods of selection of participants. Describe methods of follow-up	6-7
		(*b*) For matched studies, give matching criteria and number of exposed and unexposed	
Variables	7	Clearly define all outcomes, exposures, predictors, potential confounders, and effect modifiers. Give diagnostic criteria, if applicable	6-8
Data sources/ measurement	8*	For each variable of interest, give sources of data and details of methods of assessment (measurement). Describe comparability of assessment methods if there is more than one group	7-8
Bias	9	Describe any efforts to address potential sources of bias	8
Study size	10	Explain how the study size was arrived at	6-7
Quantitative variables	11	Explain how quantitative variables were handled in the analyses. If applicable, describe which groupings were chosen and why	8-9

Statistical methods	12	(*a*) Describe all statistical methods, including those used to control for confounding	8-9
		(*b*) Describe any methods used to examine subgroups and interactions	8-9
		(*c*) Explain how missing data were addressed	
		(*d*) If applicable, explain how loss to follow-up was addressed	
		(*e*) Describe any sensitivity analyses	
**Results**			
Participants	13*	(a) Report numbers of individuals at each stage of study—eg numbers potentially eligible, examined for eligibility, confirmed eligible, included in the study, completing follow-up, and analysed	9
		(b) Give reasons for non-participation at each stage	
		(c) Consider use of a flow diagram	
Descriptive data	14*	(a) Give characteristics of study participants (eg demographic, clinical, social) and information on exposures and potential confounders	9-11
		(b) Indicate number of participants with missing data for each variable of interest	
		(c) Summarise follow-up time (eg, average and total amount)	
Outcome data	15*	Report numbers of outcome events or summary measures over time	11-16

Main results	16	(*a*) Give unadjusted estimates and, if applicable, confounder-adjusted estimates and their precision (eg, 95% confidence interval). Make clear which confounders were adjusted for and why they were included	11-16
		(*b*) Report category boundaries when continuous variables were categorized	
		(*c*) If relevant, consider translating estimates of relative risk into absolute risk for a meaningful time period	
Other analyses	17	Report other analyses done—eg analyses of subgroups and interactions, and sensitivity analyses	11-16
**Discussion**			
Key results	18	Summarise key results with reference to study objectives	16-19

Limitations	19	Discuss limitations of the study, taking into account sources of potential bias or imprecision. Discuss both direction and magnitude of any potential bias	23
Interpretation	20	Give a cautious overall interpretation of results considering objectives, limitations, multiplicity of analyses, results from similar studies, and other relevant evidence	16-19
Generalisability	21	Discuss the generalisability (external validity) of the study results	23
**Other information**			
Funding	22	Give the source of funding and the role of the funders for the present study and, if applicable, for the original study on which the present article is based	24

*Give information separately for exposed and unexposed groups.

File S3. National reimbursement criteria for cemiplimab in cervical cancer in Poland [3]

Scope of the reimbursement program

- Cemiplimab is reimbursed within a national drug program dedicated to PD-1 inhibitors for cervical cancer.
- Reimbursement applies to cemiplimab monotherapy used in the second or third line of systemic treatment.
- Treatment is indicated after progression on platinum-based chemotherapy, with or without prior bevacizumab.
- The program includes patients with persistent, recurrent, or metastatic disease not eligible for curative surgery or radiotherapy.

General eligibility criteria

- Histologically confirmed cervical cancer of:
    - squamous-cell carcinoma,
    - adenocarcinoma,
    - adenosquamous carcinoma.
- Measurable disease according to RECIST criteria.
- Age ≥18 years.

- ECOG performance status 0–1 at treatment initiation.
- Adequate renal, hepatic, and bone marrow function.
- Absence of symptomatic central nervous system metastases.
- No uncontrolled concomitant malignancies.
- No active autoimmune disease, with predefined exceptions (type 1 diabetes, treated autoimmune hypothyroidism, psoriasis, vitiligo).
- No prior treatment with PD-1 or PD-L1 inhibitors.
- No contraindications to cemiplimab according to the summary of product characteristics.

Treatment-specific criteria

- Disease progression during or after platinum-based chemotherapy.
- Lack of eligibility for radical surgical or radiotherapeutic treatment.
- Written informed consent, including compliance with contraception requirements where applicable.

Baseline assessments required for qualification

- Histopathological confirmation of cervical cancer subtype.
- Laboratory evaluation of organ function, including:
    - complete blood count,
    - renal and hepatic function tests,
    - thyroid function tests.
- Pregnancy testing in women of childbearing potential.
- Electrocardiography.
- Baseline imaging of the chest, abdomen, and pelvis to enable objective response assessment according to RECIST.

Treatment administration

- Cemiplimab administered at a fixed dose of 350 mg intravenously every 3 weeks (Q3W).
- Treatment continued until:
    - confirmed disease progression,
    - unacceptable toxicity,

- deterioration in performance status,
- other clinical circumstances precluding further therapy, as determined by the treating physician.

Continuation of treatment

Patients previously treated with cemiplimab under alternative public funding mechanisms were eligible for continuation, provided that program eligibility criteria were met at treatment initiation.

File S4. Baseline characteristics of the enrolled patients (n=101) – additional information

		**All (n = 101)**	**irAEs (n = 34)**	**no irAEs (n = 67)**	**p value**
BMI – median (IQR)		24.6 (21.4 – 28.5)	24.4 (21.4–28.4)	24.7 (21.6–28.5)	0.62
Obesity (BMI ≥ 30.0) – n (%)		19 (18.8)	6 (17.7)	13 (19.4)	1.0
Comorbidities – n (%)	Cardiovascular	31 (30.7)	10 (29.4)	21 (31.3)	1.0
	Nephrological	26 (25.7)	7 (20.6)	19 (28.4)	0.49
	Endocrinological	16 (15.8)	6 (17.7)	10 (14.9)	0.78
	DVT	14 (13.9)	3 (8.8)	11 (16.4)	0.33
	Hypercholesterolemia	9 (8.9)	2 (5.9)	7 (10.4)	0.46
	Autoimmune	5 (4.9)	2 (5.9)	3 (4.5)	1.0
	Pulmonary	4 (4.0)	1 (3.0)	3 (4.5)	1.0
Sites of metastasis at cemiplimab initiation – n (%)	Nonregional lymph nodes	59 (58.4)	17 (50.0)	42 (62.7)	0.15
	Lungs	35 (34.7)	10 (29.4)	25 (37.3)	0.53
	Liver	24 (23.8)	7 (20.6)	17 (25.4)	0.61
	Bones	19 (18.8)	5 (14.7)	14 (20.9)	0.62
	CNS	3 (3.0)	2 (5.9)	1 (1.5)	0.42
	Other	53 (52.5)	17 (50.0)	36 (53.7)	0.76
Number of metastatic sited at cemiplimab initiation – n (%)	< 2	53 (52.5)	21 (61.7)	32 (47.8)	0.18
	≥ 2	48 (47.5)	13 (38.3)	35 (52.2)	0.18

Abbreviations: BMI – body mass index; CNS – central nervous system; DVT – deep vein thrombosis; IQR – interquartile range; irAEs – immune-related adverse events

Figure S1. Kaplan–Meier curves for A- overall survival (OS) and B- progression-free survival (PFS) stratified by programmed death-ligand 1 (PD-L1) combined positive score (CPS) status. CPS categories are presented in the legend as numeric codes: 0 = CPS-negative, 1 = CPS-positive, and 2 = CPS-unknown.

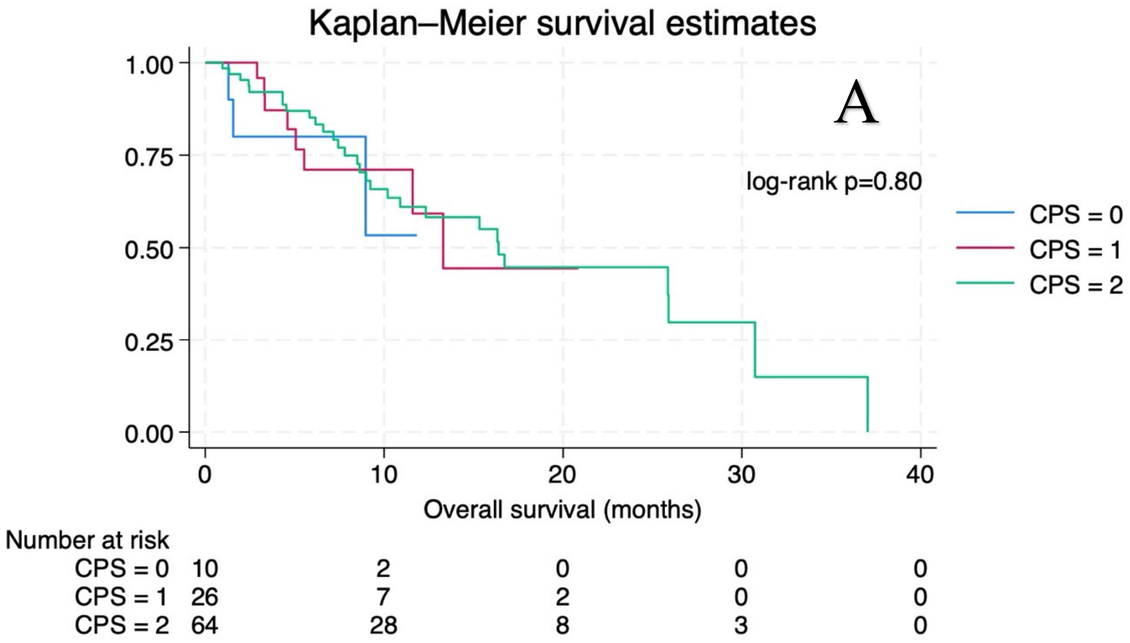

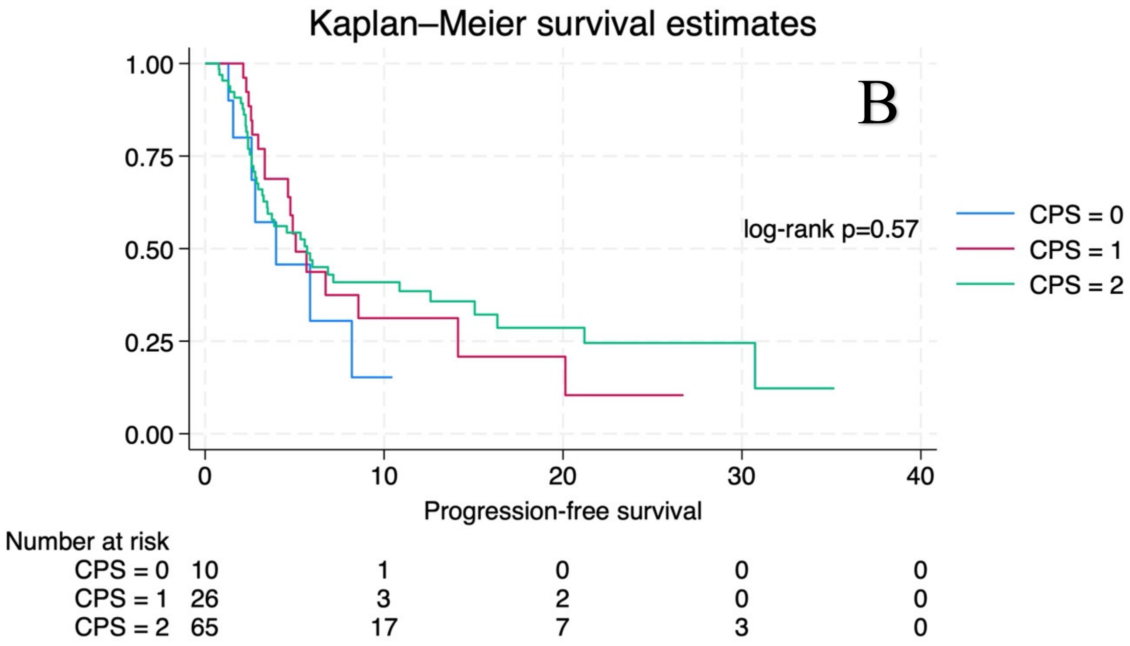

References

1. Loscalzo J, Fauci A, Kasper D, Hauser S, Longo D, Jameson J. Harrison's Principles of Internal Medicine, Twenty-First Edition (Vol.1 & Vol.2). McGraw Hill LLC. McGraw Hill / Medical; 2022.

2. Checklists - STROBE. https://www.strobe-statement.org/checklists/.

3. Program lekowy Ministerstwa Zdrowia, https://www.gov.pl/web/zdrowie/choroby-onkologiczne.
